# Community-Wide Efforts to Improve the Consumer Food Environment and Physical Activity Resources in Rural Kentucky

**DOI:** 10.5888/pcd16.180322

**Published:** 2019-01-17

**Authors:** Alison Gustafson, Margaret McGladrey, Tammy Stephenson, Janet Kurzynske, Janet Mullins, Nicole Peritore, Kathryn Cardarelli, Ann Vail

**Affiliations:** 1 Department of Dietetics and Human Nutrition, University of Kentucky, Lexington, Kentucky; 2 UK Center for Research on Violence Against Women, University of Kentucky, Lexington, Kentucky; 3 College of Social Work, University of Kentucky, Lexington, Kentucky; 4 Department of Kinesiology, University of Augusta, Augusta, Georgia; 5 Department of Health, Behavior, and Society, College of Public Health, University of Kentucky, Lexington, Kentucky

## Abstract

Community interventions to improve access to food and physical activity resources can reduce obesity rates and improve obesity-related health outcomes. We describe a Kentucky community project that consisted of collaborating with grocery store managers to improve the consumer food environment and partnering with community members to improve walking trails, bicycle racks, and other physical activity resources. We surveyed 2 random samples of community residents in 6 participating rural counties, 741 in 2016 (year 1) and 1,807 in 2017 (year 2). Fruit and vegetable intake significantly increased from year 1 (mean servings fruits, 2.71; vegetables, 2.54) to year 2 (mean servings fruit, 2.94; vegetables, 2.72). Although moderate physical activity did not change from year 1 to year 2, concern among residents about places to be physically active improved (*P* = .04). Involving community members in promoting obesity prevention programs may improve dietary intake and alleviate community concern about physical activity.

**Summary**

**What is already known about this topic?**

Several states participated in the Centers for Disease Control and Prevention High Obesity grant project. Obesity affects adults and children across a broad spectrum of geographic, socioeconomic, and racial/ethnic populations. To date communities have struggled to address how best to decrease the rates of obesity among the most marginalized populations.

**What is added by this report?**

From 2014 through 2018 a variety of nutrition and physical activity strategies were implemented across 6 counties in rural Kentucky with the goal of improving food access and resources for being physically active. We highlight the success of these programs in aiming to improving dietary intake and physical activity.

**What are the implications for public health practice?**

By understanding what community-driven nutrition and physical activity strategies are successful, other communities can develop and implement similar programs.

## Introduction

Compared with urban communities, rural communities face greater barriers to healthy eating and active living, such as limited access to food, transportation barriers, fewer sidewalks, and fewer resources for physical activity. These barriers contribute to higher rates of obesity in rural communities than in their urban counterparts (1,2). A host of factors related to geographic isolation, socioeconomic status, and lack of access to affordable healthy foods all contribute to the prevalence of obesity and poor dietary outcomes (1,3). One approach to targeting obesity is through community programs.

Recent community efforts among African American adult women in the rural South have shown significant success with improved intake of fruits and vegetables and increased physical activity (4). Another school-based intervention involving community outreach also showed improved intake of fruits and vegetables (5). Although these community efforts used individual-level approaches, such as nutrition education through face-to-face sessions and in-class sessions, they did not address the built environment as a way to improve access to healthy foods and places to be physically active. Results from previous multilevel interventions targeting both urban and rural populations (6,7) suggest that tailored community-based interventions can improve health outcomes (7). However, research is needed to understand how changing the consumer food and physical activity environments in rural communities can improve health outcomes (8).

Research focusing on interventions directed at the consumer food environment (eg, items available in grocery stores) to improve nutrition has reported using recipe samples and placing products strategically as a way to increase purchases of healthy food items (9,10). In addition, a parallel focus is needed on improving community resources for physical activity. Research shows that when people have access to safe places for physical activity, the likelihood of their engaging in physical activity increases (4). Community involvement can help determine the type and location of physical activity enhancements.

We describe a community intervention conducted among 6 rural Kentucky counties from March 2016 through May 2017 to make environmental changes to promote access to healthy food and physical activity. The primary evaluation outcomes were self-reported results of surveys of adults about their intake of fruits and vegetables and minutes of moderate physical activity engaged in between baseline in March through May of 2016 and completion from March through May 2017, one year after implementation. Our objectives were to determine the effectiveness of a community-based program by using a quasi-experimental study design to assess mean differences in dietary intake; minutes of moderate and vigorous physical activity; and community concern about obesity, healthy eating, and physical activity.

## Purpose and Objectives

Our project was funded by a cooperative agreement with the Centers for Disease Control and Prevention (CDC). Because the goal of our CDC cooperative agreement was program evaluation of development and delivery interventions at the community level, we used a quasi-experimental study design. Baseline data were collected in year 1 of the study before the intervention began, and data from follow-up surveys were collected in 2017 after completion of the intervention. To understand the key drivers of obesity and identify opportunities for obesity prevention in rural communities we selected 6 counties on the basis of US Department of Agriculture Rural Codes of 7 or higher (https://www.ers.usda.gov/data-products/rural-urban-continuum-codes/) and on the basis of an obesity prevalence of 40% or more (Clinton, Elliott, Letcher, Lewis, Logan, and Martin counties). These counties were identified as high-priority areas for intervention by the 1416 High Obesity Areas Grant Program of the Centers for Disease Control and Prevention (CDC) Division of Nutrition, Physical Activity, and Obesity (2). These counties had poverty rates ranging from 25.7% to 35.7%, food insecurity rates of 15.2% to 20.1%, and an unemployment rate ranging from 9.6% to 17.3%.

Residents’ engagement in assessing community food environment and physical activity needs and assets was facilitated by Family Consumer Science (FCS) Extension Agents in each county (11). Each agent recruited and convened a group of county stakeholders — health care providers and personnel from grocery stores, public health departments, and public libraries — in planning meetings to evaluate community needs and assets. University faculty and staff guided stakeholders in generating a list of community assets, discussing the risk factors contributing to obesity in their counties and mapping these obesity risk factors onto the identified assets. High produce costs resulting from geographic remoteness were identified as an unaddressed barrier to accessing fresh food and a contributing factor to obesity. In addition, the lack of safe and affordable resources for being physical active was another factor identified. These insights prioritized the targeting of grocery stores and farmers markets and improving resources that facilitate physical activity.

## Intervention

### Consumer food environment

The Plate it Up Kentucky Proud (PIU) social marketing campaign is a collaboration among University of Kentucky students, faculty, and staff; FCS extension agents; and the Kentucky State Department of Agriculture. As part of the PIU campaign, healthy recipes incorporating locally grown, in-season fruits and vegetables are developed by undergraduate dietetics and human nutrition students. Following taste-testing and evaluation, select recipes are prepared by FCS extension agents for further testing in the community setting.

Supermarkets with 5 to 7 cash registers were asked to participate in the PIU social marketing campaign in years 1 and 2 of the project. In each county, at least 1 store participated, and 17 stores participated in years 1 and 2 (Lewis County, 3 stores; Martin County, 2; Clinton County, 3; Logan County, 4; Letcher County, 3; Elliot County, 2). Stores with 5 to 7 cash registers were designated as supermarkets (n = 16), and stores with 8 cash registers or more (n = 1) were designated as supercenters. Evidence-based marketing strategies in the stores were implemented to heighten awareness of the PIU brand, including recipe samples offered at grocery store entrances and produce offered at check-out end caps. Additionally, children’s shopping carts, placards for grocery carts with PIU recipes and the PIU logo, and a banner of the PIU logo outside each grocery store were provided.

All farmers markets in the counties participated in PIU events during their season (May–September of years 1 and 2). Tote bags and gel packs were distributed as incentives for PIU sampling, and $5 gas cards were distributed to encourage shopping at farmers markets.

### Physical activity resources

County coalitions determined which physical activity enhancements would be best suited for their communities. Selections were wide-ranging, from Fit-Trail installations to park benches, from park bathroom renovations to water bottle–filling stations, from road striping for bicycles and pedestrians to sunshades in parks and athletic fields. These diverse actions were selected to remove barriers to physical activity. FCS Extension Agents in each county offered programs that involved the use of the enhancements, such as conducting a “bike rodeo” in a park with new benches, trash cans, water bottle–filling stations, and bike racks.

### Evaluation

Random-digit–dial surveys were conducted in years 1 and 2 for the 6-county region. A detailed description of each county and methods for sampling residents are available (11). Briefly, adult residents in all counties were called who had either land lines or cellular telephones. The random-digit–dial procedure ensured that every residential telephone line (both landline and cellular) in these Kentucky counties had an equal probability of being called. Households were screened to identify the adult primary food shopper. Primary food shopper was determined by asking the following: “Do you conduct at least 25% of the food shopping per week for your household?”. If the person responded yes, the survey continued. If the person responded no, the caller asked to speak with the primary food shopper in the household. Demographic questions assessed income level, sex, age, years of residence, and marital status.

Up to 15 call attempts were made with up to 10 scheduled callbacks to those reached at an inconvenient time. The final sample for year was 1 was 741 respondents, and for year 2, 1,807. These were 2 separate samples and were thus treated as distinct random samples. The University of Kentucky institutional review board approved this study.

### Outcome measures

The primary outcome was change in fruit and vegetable intake, measured in the survey as, “On a typical day, how many servings of fruits or vegetables do you consume?” The response options consisted of less than one serving, 1 serving, 2 servings, 3 servings, 4 servings, 5 servings, or 6 servings. These questions were previously validated among the National Cancer Institute Eating at America’s Table (12).

To capture physical activity minutes, the survey asked how often the person engaged in moderate physical activity (defined as 30 minutes of moderate activity such as walking, light jogging, gardening; 3.0 to 6.0 metabolic equivalents (METs) of energy expenditure) in minutes and then days per week. The same question was asked for vigorous physical activity (defined as 30 to 45 minutes of vigorous activity such as running, cycling, rowing; >6.0 METs of energy expenditure). These questions were taken from the National Health and Nutrition Examination Survey Physical Activity and Physical Fitness Questionnaire (https://wwwn.cdc.gov/Nchs/Nhanes/2015-2016/PAQ_I.htm).

Secondary outcomes were changes in shopping behaviors, assessed by asking where and how often respondents shopped at the following types of food venues: supercenters, supermarkets, and farmers markets or community-supported agriculture gardens. Response options were 2 or more times per week, once per week, 2 to 3 times per month, once per month, a few times per year, never, and don’t know. These response options were collapsed to create categorical variables of 2 to 3 times per month, including once per month, a few times per year, and at least once per week. These questions have been used among rural residents in Kentucky and North Carolina (11).

Our study assessed whether there was an increase in levels of concern about obesity, healthy eating, and physical activity among surveyed participants after being exposed to the intervention for more than a year. From a 2016 survey among those participating in Department of Agriculture Cooperative Extension Service programs in the same Kentucky counties we studied, we determined that 1,000 to 9,000 families were reached via Extension Service efforts related to information about accessing healthy food, and 0 to 7 physical activity environmental changes were implemented in the counties. Therefore, to determine whether there was an increase in overall community concern about healthy eating, obesity, and physical activity, we asked several questions. To assess levels of concern, respondents were asked whether obesity, healthy eating, and physical activity in their community were a concern. Response options were not at all a concern, minor concern, moderate concern, serious concern, and don’t know. To assess these changes from year 1 to year 2, we used Fisher’s exact tests for categorical variables and *t* tests to assess changes in mean servings of fruits and vegetable consumed and mean minutes and days per week of moderate and vigorous physical activity, adjusted for age, income, race/ethnicity, and sex. Stata 14.0 (StataCorp LP) was used in all analyses, weighted for the sample size in each county (13).

## Results

From year 1 to year 2, the mean number of servings per day of fruit increased significantly from 2.71 to 2.94 (*P* = .03), and the mean number of servings per day of vegetables increased from 2.54 to 2.72 (*P* = .04)(Table 1). No significant change occurred from year 1 to year 2 in shopping frequency at primary type of food store. However, there was an increase in mean frequency of shopping at farmers markets, from 7% shopping at farmers markets once a week in year 1 to 12% in year 2.

**Table 1:** Demographic Characteristics and Changes in Shopping, and Dietary Habits Among Community Residents (N = 2,548) in Rural Counties With High Prevalence of Obesity, Kentucky 2016–2017^a^

Characteristic	Year 1 (n = 741)	Year 2 (n = 1,807)
**Female sex**	75 (555)	73 (1,319)
**Participant in Supplemental Nutrition Assistance Program**	13 (96)	19 (343)
**Education**		
High school graduate or GED	27 (200)	30 (542)
Some college	22 (163)	23 (415)
**Married**	64(474)^b ^	57(1,029)^b^
**Dietary habits**		
Servings of fruit/d, mean (SD)	2.71 (2.26)^b^	2.94 (2.72)^b^
Servings of vegetables, mean (SD)	2.54 (2.35)^c^	2.72 (2.25)^c^
**Physical activity, min/d, mean (SD)**		
Moderate activity	131 (0.41)	128 (0.43)
Vigorous activity	99.92 (0.64)	113 (0.72)
**Physical activity, days/wk, mean (SD)^d^**		
Moderate activity	4.6 (2.05)	4.7 (2.52)
Vigorous activity	4.38 (1.92)	4.3 (1.09)
**Type of store for primary shopping^c^**		
Supercenter	85(630)	85 (1,535)
Supermarket	65(481)	63(1,138)
**Frequency of shopping at supercenter^e^**		
2–3 times per month	24(178	28 (506)
1 time per week	32 (237)	30 (542)
**Frequency of shopping at supermarket^e^**		
2–3 times per month	23 (176)	24 (434)
1 time per week	26 (182)	26 (470)
**Fruit and vegetable community shopping (farmers market, CSA garden)**		
2–3 times per month	10 (74)	10 (19)
1 time per week	7 (52)^c^	12 (217)^c^
**Distance from farmers market, miles, mean (SE)**	9 (.34)	9 (.21)

Abbreviations: CSA, community supported agriculture; GED, general equivalency degree; SD, standard deviation; SE, standard error.

a Values are number (percentage) unless otherwise indicated. Percentages may not total 100 because of rounding. Totals in some categories may not correspond to overall totals because of nonresponders in some categories of questions.

b Significance of change from year 1 to year 2, *P *= .03.

c Significance of change from year 1 to year 2, *P* = .04.

d Moderate physical activity = 3.0 to 6.0 metabolic equivalents (METs) of energy expenditure; vigorous physical activity = >6.0 METs.

e A midsize supermarket has 5 to 7 cash registers; a supercenter has at least 8 cash registers.

Our analysis of the variables measuring community concern about obesity, healthy eating, and awareness of PIU indicated that levels of concern about obesity, healthy eating, and physical activity, changed significantly from year 1 to year 2 (Table 2).

**Table 2:** Concern Among Community Members (N = 2,548) About Social Marketing Changes on Obesity, Healthy Eating, and Physical Activity from Year 1 to Year 2 in Rural Counties With High Prevalence of Obesity, Kentucky 2016–2017^a^

Area of Concern	Year 1 (n = 741)^b^	Year 2 (n = 1,807)	*P* Value^c^
**Obesity**			
Not at all a concern	15 (2)	110 (6)	.02
Somewhat a concern	600 (81)	947 (52)	
Serious concern	111 (15)	750 (41)	
**Healthy eating**			
Not at all a concern	22 (3)	116 (6)	.03
Somewhat a concern	615 (83)	1,061 (58)	
Serious concern	110 (14)	630 (34)	
**Physical activity**			
Not at all a concern	22 (3)	156 (8)	.04
Somewhat a concern	630 (85)	1,122 (62)	
Serious concern	77 (11)	529 (29)	

a Values are number (percentage). Percentages may not total 100 because of rounding.

b Values for obesity and physical activity do not total 741 because of 5% nonresponders.

c Change from year 1 to year 2.

## Implications for Public Health

Our program targeting small and mid-sized rural supermarkets and farmers markets improved dietary intake of fruits and vegetables and shopping frequency at farmers markets. Previous research indicated that community interventions were modestly successful in addressing key health outcomes, including via social marketing campaigns (14) and taste-testing, which our results support. In addition to these established marketing strategies, PIU addressed the communities’ food retail infrastructure. Recipe samples and placement of healthy items at check-out counters led to purchase of healthier food (15), as did signage on grocery carts. These findings suggest that the enhancements to the consumer food environment (recipe samples, product placement, signage) combined with social marketing approaches were effective in improving fruit and vegetable intake in rural communities.

Our findings should be interpreted cautiously. Because we used a quasi-experimental study design, no causation can be established. Data on costs were not collected to determine cost-effectiveness of our strategies (8). Much of the physical activity infrastructure was new at the time of the second survey, and programming around the infrastructure was still limited. Another limitation was the difference in sample size between years 1 and 2 generated by the random-digit–dial method. The difference may be related to the possibility that residents became familiar with the program by year 2 and were more willing to respond to the survey team. Nevertheless, these results suggest a role that community residents and store owners can play in improving the rural consumer food environment.

Our findings suggest that involving community members and grocery store owners was key in improving the community food environment in rural counties. Social marketing programs such as PIU appear to be useful in raising awareness and concern about healthy eating and obesity in small, rural communities with limited consumer food options. Campaigns like PIU can “blanket” the consumer food environment of rural counties and aid in improving access to healthy foods.
