# Erratum, Vol. 16, January 17, 2019, Release

**DOI:** 10.5888/pcd16.180322e

**Published:** 2019-02-14

Because of a technical production error, tables in this article were inadvertently switched with those of another article. We corrected this error on January 18, 2019, and the article appears online at https://www.cdc.gov/pcd/issues/2019/18_0322.htm. We regret any confusion or inconvenience that this error may have caused.

